# Histopathological Outcomes of Thy3 Thyroid Nodules: Malignancy Risk in an Indeterminate Cytology Cohort

**DOI:** 10.7759/cureus.106622

**Published:** 2026-04-07

**Authors:** Paraskevi Karamitsou, Carlos Galan, Anant Patel, Ananth Vijendren, George Mochloulis, Panagiotis Dimitriadis

**Affiliations:** 1 Department of Ear, Nose and Throat (ENT), Lister Hospital, East and North Hertfordshire Teaching NHS Trust, Stevenage, GBR; 2 Department of Ear, Nose and Throat (ENT), University of Navarra Clinic, Madrid, ESP

**Keywords:** fine needle aspiration, thy3 thyroid nodule, thyroid cancer, thyroid cytology, thyroid histology

## Abstract

Introduction: In our centre, we observed a higher than nationally reported malignancy rate among indeterminate thyroid nodules. As a result, we adopted a practice of offering diagnostic hemithyroidectomy to patients with Thy3a cytology. This study aims to evaluate the malignancy rate of indeterminate thyroid nodules following this change in practice.

Methods: We conducted a retrospective study of patients with Thy3 thyroid nodules who underwent thyroidectomy at our centre between January 2022 and December 2023. We recorded and analysed patients’ demographics, nodule size, and histopathological outcomes.

Results: During the study period, 262 thyroidectomies were performed, of which 136 (51.9%) had a pre-operative Thy3 cytology. The majority of patients were females (N=97, 71.3%), and the average age was 51.4 years. The overall malignancy rate was 24.3%. In the Thy3a group (N=109), the malignancy rate was 19.3%, and the nodule size in the benign group was larger (38.1mm versus 31.7mm). In the Thy3f group (N=27), the malignancy rate was 44.4%, and the nodule size did not differ significantly between the groups (24.7mm versus 25.2mm).

Conclusion: Almost every second patient with Thy3f cytology and approximately one in five patients with Thy3a cytology were diagnosed with thyroid cancer. Given the significant variation between centres, we recommend that individual endocrine units assess their local malignancy rates to better inform discussions with patients.

## Introduction

The prevalence of thyroid nodules in the general population is notably high. High-resolution ultrasonography can detect one or more thyroid nodules in approximately 60% of adults [1]. Of those detected, about 5% are finally proven to be malignant [1].

In the United Kingdom (UK), thyroid cancer ranks as the 20th most common cancer [2,3]. Over the last decade, thyroid cancer incidence rates have increased by 61% in females and by 64% in males [2,3]. This rise in incidence, partly, reflects the increasing detection of asymptomatic cases. The discovery of subclinical nodules has been facilitated by the widespread use of medical imaging techniques and improved access to healthcare services.

Ultrasound is the principal imaging technique used for the initial thyroid nodule assessment. Ultrasound‐guided fine-needle aspiration cytology (FNAc) is considered the gold standard for investigating thyroid nodules [4]. The main objective of diagnosing thyroid nodules is to establish a cost-effective process that safely identifies malignancy while minimising overtreatment [5]. Management of the patients with thyroid nodules may vary (e.g. surgical intervention, active surveillance, discharge) depending on ultrasonography and thyroid cytology findings, which are commonly discussed in a multidisciplinary team (MDT) setting [6].

The British Thyroid Association (BTA)/Royal College of Pathologists (RCPath) Thy1-Thy5 system is the most widely used thyroid cytology classification in the UK [7,8]. Worldwide, other thyroid cytology reporting systems are in use; among these, the Bethesda system is the most widely adopted and correlates closely with the Thy1-5 system [9-11]. All these systems include a category for thyroid nodules that are not definitively diagnosable through cytology. In the Thy3 category, the nature of the lesion cannot be determined solely by FNAc and is therefore considered indeterminate [8]. The Thy3 category is further divided into Thy3a (Bethesda 3), suggesting cellular atypia, and Thy3f (Bethesda 4), indicating a follicular lesion. National data indicate that the risk of malignancy is approximately 5-15% in Thy3a thyroid nodules and 15-30% in Thy3f nodules [8]. Follicular neoplasms require surgical excision to evaluate for capsular and/or vascular invasion, which is necessary for determining malignancy [12]. According to BTA, surveillance ultrasound assessment with or without FNA is offered to a patient with Thy3a thyroid nodule [7]. If repeat cytology remains Thy3a, an MDT discussion is required for patient management. Diagnostic hemithyroidectomy is recommended for the Thy3f thyroid nodules [7].

In our centre, the thyroid MDT would typically recommend diagnostic surgery for patients with Thy3a cytology and associated risk factors, such as family history of thyroid cancer, history of neck irradiation, increase in nodule size over time, presence of nuclear atypia on cytology and for every patient with Thy3f cytology. We observed a higher-than-average malignancy rate for patients with indeterminate thyroid cytology in our centre [13]. Consequently, we started offering diagnostic hemithyroidectomy to all patients with Thy3a cytology as an alternative to conservative management or discharge. This study primarily aims to evaluate malignancy risk in surgically treated Thy3 thyroid nodules at our centre over a two-year period following this change in clinical practice. Secondary objectives are to compare malignancy rates between Thy3a and Thy3f nodules and to assess the impact of broader patient selection on observed malignancy rates and clinical decision-making.

## Materials and methods

The study was conducted in accordance with the Declaration of Helsinki and all its subsequent amendments. This retrospective observational study examined cases of Thy3 thyroid nodules (Bethesda 3 and 4) that were operated on in the Department of Otolaryngology-Head and Neck Surgery of Lister Hospital (East and North Hertfordshire Teaching NHS Trust, Stevenage, UK) between January 2022 and December 2023. Table 1 presents the correlations between the Thy1-5 and Bethesda systems.

**Table 1:** Correlation between Thy1-Thy5 and Bethesda systems for reporting thyroid cytology

Thy1-Thy5 System	Bethesda System
Thy1 - non-diagnostic	1 - non-diagnostic
Thy2 - non-neoplastic	2 - benign
Thy3a - atypia	3 - atypia of undetermined significance or follicular lesion of undetermined significance
Thy3f - follicular neoplasm	4 - follicular neoplasm or suspicious for a follicular neoplasm
Thy4 - suspicious for malignancy	5 - suspicious for malignancy
Thy5 - malignancy	6 - malignancy

Since this is a retrospective study with no intervention, approval from the Institutional Review Board (IRB) is not required.

All patients with preoperative Thy3 cytology were eligible for inclusion. Patients with repeat FNAc were included in the study if at least one of the samples was classified as Thy3. Those who underwent a complete thyroidectomy following an initial diagnostic hemithyroidectomy were only counted once. Patients with non-diagnostic (Thy1), benign (Thy2), suspicious for malignancy (Thy4), or malignant (Thy5) cytology were excluded from the study.

A data extraction template was created using Microsoft Office Excel (version 16, Microsoft Corp., Redmond, WA, USA). Variables collected included patient demographics (age, gender), surgical details (hemithyroidectomy, total thyroidectomy, completion thyroidectomy), laterality (left, right, bilateral), nodule size (maximum dimension), and final histopathological diagnosis. The histopathological outcomes were compared with the preoperative FNAc to assess the risk of malignancy in the Thy3a and Thy3f groups. All included cases had complete datasets for the variables analysed, and no missing data were identified.

All FNAc samples were reported according to the BTA/RCPath Thy1-5 classification system. Cytology outcomes were independently reported by three pathologists, with additional review by a senior pathologist specialising in thyroid cytology to minimise inter-observer variability and ensure diagnostic consistency. Histopathological examination of surgical specimens was conducted in accordance with standard departmental protocols. Cases with ambiguous findings were reviewed within the MDT setting to achieve diagnostic consensus.

Statistical analysis was performed using Microsoft Office Excel, and all data were expressed as frequencies with corresponding percentages. Comparisons between groups were performed using contingency tables.

## Results

A total of 262 thyroidectomies were performed between January 2022 and December 2023. Among these patients, a pre-operative Thy3 cytology was identified in 136 cases (51.9%). The average age of the patients was 51.4 years. Of the 136 patients, 97 (71.3%) were females. The patients were further classified into subgroups, Thy3a and Thy3f, and a subgroup analysis was conducted to determine malignancy rates and to explore potential correlations with other factors, such as demographic characteristics, the size of the thyroid nodules, and the presence of nuclear atypia. Data regarding the malignancy rates of Thy3 thyroid nodules are summarised in Table 2.

**Table 2:** Malignancy rates and demographic characteristics of Thy3 thyroid nodules and subgroups (Thy3a and Thy3f)

Characteristic	Benign	Malignant
Thy3 (Bethesda 3 and 4)		
Number of patients, n (%)	103 (75.7%)	33 (24.3%)
Average age (range), years	52.9 (18-84)	49.9 (20-84)
Average nodule size, mm	36.5	29.2
Female	78	19
Male	27	12
Thy3a (Bethesda 3)		
Number of patients, n (%)	88 (80.7%)	21 (19.3%)
Average age (range), years	52.6 (28-80)	48.1 (21-75)
Average nodule size, mm	38.1	31.7
Female	73	14
Male	19	3
Thy3f (Bethesda 4)		
Number of patients, n (%)	15 (55.6%)	12 (44.4%)
Average age (range), years	54 (18-84)	53.9 (20-84)
Average nodule size, mm	24.7	25.2
Female	6	8
Male	7	6

During the study period, 37 patients with Thy3a cytology were discharged following MDT discussion, while all remaining Thy3a cases (109 patients) proceeded to surgery.

The overall malignancy rate for the Thy3 (Bethesda 3 and 4) cytology was 24.3%. Patients with malignant final histology had an average age of 49.9 years (ranging from 20 to 84 years), while those with benign histology had an average age of 52.9 years (ranging from 18 to 84 years). In terms of thyroid nodule size, patients with malignant histology had an average nodule size of 29.2mm (maximum dimension). Benign nodules were found to be larger, with an average nodule size of 36.5mm.

The Thy3a (Bethesda 3) subgroup consisted of 109 patients, comprising 87 females (79.8%) and 22 males (20.2%), with a malignancy rate of 19.3%. Among the patients with malignant final histology, the average age was 48.1 years (ranging from 21 to 75 years), while those with benign disease had an average age of 52.6 years (ranging from 28 to 80 years). The average size of malignant nodules measured 31.7mm, whereas for benign nodules it was 38.1mm. The presence of nuclear atypia was reported in five patients, three of whom (60%) were diagnosed with thyroid cancer.

The Thy3f (Bethesda 4) subgroup included 27 patients (14 females (51.9%) and 13 males (48.1%)), who exhibited a malignancy rate of 44.4%. The average age of patients diagnosed with thyroid cancer was 53.9 years (ranging from 20 to 84 years). Patients with benign thyroid disease had an average age of 54 years (ranging from 18 to 84 years). Regarding thyroid nodule size, no significant difference was observed between malignant and benign cases (25.2mm versus 24.7mm, respectively).

On histopathological examination, 18 patients (54.6%) were diagnosed with papillary thyroid carcinoma (PTC), 14 (42.4%) with follicular thyroid carcinoma (FTC), and one patient (3.0%) with medullary thyroid carcinoma (MTC).

## Discussion

The BTA/RCPath Thy1-5 system for thyroid cytology is widely used in the UK [7,8]. Both Thy3 and Thy4 categories are considered indeterminate. Nationally reported malignancy rates are 5-15% for Thy3a nodules and 15-30% for Thy3f nodules [8]; however, substantial variability has been observed between thyroid centres.

We found that the overall malignancy rate for operated Thy3 thyroid nodules was 24.3%. Prior to broadening patient selection and routinely offering diagnostic hemithyroidectomy for Thy3a cytology, the malignancy rate for Thy3 nodules at our centre was 33.9% [13]. This observed reduction of 28.3% is likely attributable to the larger denominator, resulting from the change in our practice of performing diagnostic hemithyroidectomies more frequently for Thy3a cytology. Our current results are consistent with the findings of Pereira [14], Alexander et al. [15], and Mihai et al. [16], who reported overall malignancy rates of 22.2%, 24.7%, and 25% for Thy3 nodules, respectively. The nationally reported figure for Thy3 malignancy rate by the British Association of Endocrine and Thyroid Surgeons Audit was 27.8% [17]. On the other hand, several thyroid centres have observed a higher overall malignancy rate for patients undergoing surgery for Thy3 nodules, ranging from 30.7% to 40% [4,18,19].

In our study, Thy3a nodules were found to be less likely to be cancerous compared to Thy3f (19.3% versus 44.4%, respectively). Research by Nair et al. [4] and Pereira [14] found no significant differences in malignancy rates between patients with Thy3a and Thy3f nodules. In contrast, other analyses have suggested a stronger association between Thy3a nodules and cancer compared to Thy3f nodules (33% versus 11%, respectively) [20]. These findings highlight the considerable variability in malignancy rates in Thy3 thyroid nodules. Several factors, including staff experience, inter-observer variability, patients’ demographics, patient selection criteria for surgery, and the prevalence of thyroid cancer, may account for the varying malignancy rates associated with indeterminate cytology results [21].

The average size of thyroid nodules did not differ significantly between malignant and benign cases, and no clear relationship was observed between increasing nodule size and malignancy risk [22]. The mean age of patients with Thy3 nodules who were ultimately diagnosed with thyroid cancer was 49.9 years, slightly lower than that of patients with benign disease (52.9 years). Age patterns of thyroid cancer differ between males and females. In females, incidence peaks between 40 and 44 years, whereas in males, rates gradually increase, peaking between 70 and 74 years [2,3].

According to existing literature, 90% of all thyroid cancers are differentiated, with PTC being the most common histological type, followed by FTC [23]. Other types, including MTC, anaplastic, and poorly differentiated thyroid cancers, are rare [24]. FNAc samples exhibiting nuclear atypia have been associated with higher malignancy risk. A meta-analysis by Valderrabano et al. demonstrated that nuclear atypia is a significant predictor of malignancy in cytologically indeterminate thyroid nodules [25], a finding confirmed in our study.

Limitations

This is a retrospective observational study, so subject to numerous biases, with a lower level of evidence compared with a well-designed prospective study. Our small cohort of 136 patients from a single institution is another limiting factor, although it is comparable to published studies from other thyroid centres. The lack of correlation between cytology/histopathological outcomes and patients’ risk factors is also a limitation. Additionally, the absence of more advanced statistical analysis, including multivariate assessment, should be acknowledged as a limitation, as it limits the ability to adjust for potential confounding variables. The change in our practice during the study period - offering surgery to all Thy3a cases - introduces potential selection bias. On the positive side, we did not encounter any missing data. In terms of thyroid cytology, outcomes were reported by three pathologists, while a senior pathologist specialising in thyroid cytology reviewed the samples, helping to minimise inter-observer variability.

## Conclusions

In our study, approximately one in four patients with Thy3 thyroid nodules was diagnosed with thyroid cancer. Evidence from other centres in the UK shows significant variability in malignancy rates among Thy3 thyroid nodules. We recommend regular auditing of Thy3 malignancy rates across different thyroid units. This will enable their MDTs to provide better-informed recommendations for the benefit of the patients. Further research is required to identify additional tools that can enhance preoperative diagnostic accuracy and reduce unnecessary thyroid surgeries.
